# Heritability estimates of the Big Five personality traits based on common genetic variants

**DOI:** 10.1038/tp.2015.96

**Published:** 2015-07-14

**Authors:** R A Power, M Pluess

**Affiliations:** 1 MRC Social Genetic & Developmental Psychiatry Centre, Institute of Psychiatry, Psychology & Neuroscience, King's College London, London, UK; 2 Department of Biological and Experimental Psychology, School of Biological and Chemical Sciences, Queen Mary University of London, London, UK

## Abstract

According to twin studies, the Big Five personality traits have substantial heritable components explaining 40–60% of the variance, but identification of associated genetic variants has remained elusive. Consequently, knowledge regarding the molecular genetic architecture of personality and to what extent it is shared across the different personality traits is limited. Using genomic-relatedness-matrix residual maximum likelihood analysis (GREML), we here estimated the heritability of the Big Five personality factors (extraversion, agreeableness, conscientiousness, neuroticism and openness for experience) in a sample of 5011 European adults from 527 469 single-nucleotide polymorphisms across the genome. We tested for the heritability of each personality trait, as well as for the genetic overlap between the personality factors. We found significant and substantial heritability estimates for neuroticism (15%, s.e.=0.08, *P*=0.04) and openness (21%, s.e.=0.08, *P*<0.01), but not for extraversion, agreeableness and conscientiousness. The bivariate analyses showed that the variance explained by common variants entirely overlapped between neuroticism and openness (*rG*=1.00, *P* <0.001), despite low phenotypic correlation (*r*=−0.09, *P* <0.001), suggesting that the remaining unique heritability may be determined by rare or structural variants. As far as we are aware of, this is the first study estimating the shared and unique heritability of all Big Five personality traits using the GREML approach. Findings should be considered exploratory and suggest that detectable heritability estimates based on common variants is shared between neuroticism and openness to experiences.

## Introduction

The Big Five personality traits model is one of the most established and recognized approaches to describe and measure individual differences in personality,^1^ and includes openness to experience, conscientiousness, extraversion, agreeableness and neuroticism. Although openness captures imagination and intellectual curiosity, conscientiousness refers to carefulness and organizational ability. Extraversion is defined by positive emotions, such as gregariousness and the tendency to seek out stimulation. Neuroticism includes negative emotions, such as anxiety and depression, and is commonly defined as emotional instability, and agreeableness describes an individual's level of cooperativeness and compassion. Although the Big Five are conceptually independent from each other, most studies suggest that there are small-to-moderate associations between them (estimates ranging from *r*=0.00 to 0.40).^2, 3, 4^

According to twin studies, around 40–60% of the variance in the Big Five is heritable,^5, 6, 7^ with some overlap in heritability between personality traits themselves.^8^ For example, it has been found that a general heritable component is significantly associated with all five personality traits (estimates ranging from *r*=0.15 to 0.55).^9^ More recently, molecular studies have also attempted to dissect the underlying heritability of personality. Several genome-wide association studies have been conducted on the Big Five or other measures of personality.^10, 11, 12, 13, 14, 15, 16^ These studies have broadly been unsuccessful in identifying the underlying genetic variants for personality, suggesting variants of small individual effects or low frequency (<0.01) may have a role and that much larger samples sizes are required in order to identify these. However, more recently, genomic-relatedness-matrix residual maximum likelihood (GREML) analysis has provided new insight into the genetic architecture of personality. GREML works by looking at how very low levels of relatedness, as determined from number of shared variants across the genome, account for similarity in phenotype across traditionally unrelated individuals. In other words, GREML allows the estimation of the total genetic heritability of a trait by taking into account all gene variants available in a data set, without identifying the specific gene variants making up this heritability. Recent GREML studies of different personality traits have been able to confirm underlying genetic heritability. For example, in a sample of 12 000 unrelated individuals, common single-nucleotide polymorphisms (SNPs) accounted for 6% of the variance in neuroticism and 12% in the case of extraversion.^17^ The only other study that we are aware of reporting GREML estimates for personality traits found that genetic variants explained 7% of the variance in harm avoidance, 10% in novelty seeking and 8% in persistence (but no variance in reward dependence) in a sample of 8000 individuals.^18^ However, no study as of yet explored the Big Five in its entirety within the same sample. More importantly, existing studies did not test for overlap in genetic heritability between personality traits. Testing for genetic overlap between personality traits based on molecular evidence will not only provide us with estimates of the genetic specificity underlying each personality trait but also contribute to the on-going debate regarding the potential existence of a ‘general factor of personality', given that several twin studies reported a general common genetic factor underlying all five personality traits^9, 19^ (although other research suggests that evidence for a general factor is weak^20^).

Furthermore, such findings may inform us about the probability for success of future studies aimed at identifying specific gene variants associated with the Big Five personality traits. Should there be substantial overlap between genetic estimates of heritability of the different personality traits, it may be rather difficult for future genome-wide association studies, even those featuring large meta-analytic approaches, to identify specific gene variants for specific personality traits.

In the current study, we use genome-wide common variants to estimate the unique and shared heritability of the Big Five personality traits in a UK sample of 5011 in an effort to illuminate the underlying genetic architecture of personality and validate the estimates from twin and adoption studies.

## Materials and methods

### Sample

Data are taken from the 1958 National Child Development Study (NCDS).^21^ The NCDS is a continuing, multidisciplinary longitudinal British birth cohort study. It began when data were collected on 18 558 babies born in Great Britain (England, Scotland and Wales) in 1 week in 1958. To date, there have been seven attempts to trace all members of the birth cohort. The follow-ups were undertaken when the cohort members were aged 7, 11, 16, 23, 33, 42, 46, 50 and 55 years. Over the years, information has been gathered from a number of sources (that is, parents, schools, cohort members, doctors and medical records). Detailed information on ethics approval and informed consent across the different data collection waves is available.^22^

Our sample was a combination of three publically available genotyping efforts based on different subsamples of the NCDS: the Wellcome Trust Case Control Consortium's (WTCCC) wave 1 and 2 controls,^23^ and the Type 1 Diabetes Genetics Consortium (T1DGC) study.^24^ WTCCC1 and -2 control samples had a large amount of overlapping individuals, and here we preferentially used the WTCCC2 data due to its increased genomic coverage. Despite having a small number of additional individuals genotyped on Affymetrix platforms (Santa Clara, CA, USA), the restriction to only those in the WTCCC studies who were genotype on Illumina platforms (San Diego, CA, USA) was to allow for compatibility with the T1DGC study.

At the 50 years sweep, 9790 (52.8%) of the original 18 558 members of the Birth Cohort provided data on personality traits: 6.7% had died, 7.4% had emigrated, 6.5% refused and 26.6% could not be contacted. Of the remaining 9790, another 349 did not complete the personality questionnaire.

### Genetic data

In total, the WTCCC1 sample contributed 114 individuals genotyped on the Illumina Human Hap 550 K v1.1 platform. The WTCCC2 sample contributed 2922 individuals genotyped on the Illumina Human 1M platform. The T1DGC sample consisted of 2592 individuals on the Illumina Human Hap 550 K v3.0 platform. Quality control was performed in Genome-wide Complex Trait Analysis software.^25^ After merging data from across all three samples, we removed SNPs with high missingness (>5%) to account for the different coverage across genotyping platforms. Further, SNPs were removed based on minor allele frequency (<0.01), Hardy–Weinberg equilibrium (*P*<0.00005) and individual missingness (>0.05). We also removed 105 individuals as outliers on ancestry-informative principal components, and 48 due to shared relatedness >0.05.

### Measures

At age 50 years, cohort members were administered a ‘Big Five' personality traits questionnaire using 50 items from the International Personality Item Pool (IPIP).^26^ Each of the five personality traits were measured with 10 statements (for example, for conscientiousness: ‘am always prepared', ‘pay attention to details' for extraversion: ‘I feel comfortable around people', ‘I am the life of the party' for agreeableness: ‘I am interested in people', ‘I take time out for others' for emotional stability (that is, neuroticism): ‘I get stressed out easily', ‘I often feel blue' for imagination/intellect (that is, openness to experience): ‘I am full of ideas', I have a vivid imagination') rated by cohort members on a five-point scale ranging from ‘1=very inaccurate' to ‘5=very accurate'. Internal consistency was good with alpha=0.77 for conscientiousness, alpha=0.87 for extraversion, alpha=0.81 for agreeableness, alpha=0.88 for neuroticism and alpha=0.78 for openness. The IPIP personality traits correlate highly with similar measures, including the NEO Personality Inventory Revised (NEO-PI-R):^1^ IPIP emotional stability and NEO-PI-R neuroticism (*r*=0.82), IPIP extraversion and NEO-PI-R extraversion (*r*=0.77), IPIP imagination/intellect and NEO-PI-R openness to experience (r=0.79), IPIP agreeableness and NEO-PI-R agreeableness (*r*=0.70) and IPIP conscientiousness and NEO-PI-R conscientiousness (*r*=0.79).^27^ For the purpose of consistency in terminology, we use ‘neuroticism' when referring to the reflected IPIP scale ‘emotional stability', and ‘openness to experiences' when referring to the IPIP scale ‘imagination/intellect'.

### Statistical analysis

We performed GREML analysis to estimate the proportion of variance in personality explained by the genotyped SNPs, as described elsewhere using Genome-wide Complex Trait Analysis software.^25^ GREML analyses look at the proportion of shared SNPs across the genome between each possible pair of individuals to calculate an estimate of relatedness, and compare this genomic similarity with phenotypic similarity to estimate its heritability. Five ancestry-informative principal components were included as covariates to account for population stratification, as were subsample (in order to account for chip effects) and sex. A univariate analysis of each personality trait was performed, as well as bivariate analyses of the overlapping heritability between traits.

## Results

After quality control for genotype data and presence of personality measures, between 4855 and 4924 individuals remained for each analysis. The summary statistics for the Big Five personality measures is outlined in Table 1. Bivariate correlations between the five personality traits ranged from *r*=0.09 to 0.41. Sex was significantly associated with all personality traits, except openness. Genetic study (that is, chip effects) was not significantly associated with personality measures.

After quality control, 527 469 SNPs remained for analysis. We found a significant heritability estimate for openness to experiences (*P*=0.005), with 21% of the variance between individuals explained by the genotyped common variants included in this study, and for neuroticism (15%, *P*=0.04). All other personality traits had nonsignificant heritability estimates. Our bivariate GREML analyses showed that for these two significantly heritable traits, there was a highly significant genetic correlation of *rG*=1.00 (s.e.=0.50, *P*=0.0002). (The raw correlation estimate was *rG*=1.25. Given that estimates of bivariate correlations in Genome-wide Complex Trait Analysis software are not restricted to a range between −1 and 1, the correlation estimate of 1.25 can be interpreted as a correlation of 1 and was therefore capped at *rG*=1.00.) See Table 2 for the GREML results.

## Discussion

Our analysis adds to the growing body of work seeking to understand the genetic underpinnings of personality. Here we used molecular data to replicate the heritable component to neuroticism and openness to new experiences, as previously reported in twin studies. The proportion of variance explained for by common variants in our sample should be smaller than the estimates of heritability from twin studies, as there is poor coverage of structural or rare variation in the genome using SNPs (GREML estimates generally tend to be half of twin-study estimates^28^). That our findings account for over 15% of the variance in neuroticism and 21% of openness, compared with 40–60% from twin studies,^5, 6, 16^ suggests that common variants account for about a quarter of the causal genetic variation. The proportion for the other Big Five personality traits is much lower and nonsignificant, perhaps suggesting a different distribution of causal variant frequencies such as rare variants or non-additive genetic and environmental factors that inflate estimates of heritability in twin studies, besides the more obvious reasons of potentially insufficient sample size and phenotype measures that are not very precise. However, our findings are generally in line with other GREML analyses of personality,^17, 25^ though they perhaps show slightly greater variation in the estimates range. It is worth noting that the large standard errors around the negative findings suggest increased sample size may identify a low but significant level of heritability.

Importantly, despite moderate phenotypic correlation, neuroticism and openness showed a large overlap on a genetic level, perhaps lending some suggestive empirical support to findings from twin studies regarding the existence of a heritable general factor of personality,^9, 19^ even though in the current study this only seems to apply to neuroticism and openness on the basis of common genetic variants.

This study has several limitations, the main one being limited sample size (as suggested by the large s.e.) and a relatively short measure of the Big Five personality traits with only 10 items per trait. Samples were also combined across three genome-wide association study data sets on different chips, introducing possible confounding effects. The strengths of the study are that all Big Five personality measures were available in a large population cohort, avoiding the ascertainment biases of a clinical sample. The personality measures were also collected in adulthood, where they should remain more stable. This should allow for heritability estimates that reflect those of the general population.

As far as we are aware of, this is the first GREML study including all five of the Big Five personality traits providing new insight into the underlying genetic architecture of personality. Both personality traits identified with a heritable component in our analysis of common variants, openness and neuroticism, were found to have a large genetic despite a low phenotypic correlation, suggesting that at least some of the genetic basis of the different personality traits may be shared across traits. However, the reported findings will have to be replicated. Future studies aimed at the investigation of the genetic architecture of personality should feature large samples (or multiple large samples featuring a meta-analytic approach) with high-quality measures of the Big Five personality traits in order to test for specific and shared genetic heritability of the different personality traits.

## Figures and Tables

**Table 1:** Descriptive statistics and unadjusted bivariate associations for personality traits

*Personality trait*	N	*Mean (s.d.)*	*Gender*	*1*	*2*	*3*	*4*
Extraversion	4922	29.2 (0.09)	0.04**	—			
Agreeableness	4917	36.8 (0.08)	0.34**	0.35***	—		
Conscientiousness	4855	33.8 (0.08)	0.10**	0.15***	0.27***	—	
Neuroticism	4924	28.6 (0.10)	0.13**	−0.24***	−0.05***	−0.20***	—
Openness	4885	32.4 (0.07)	−0.02	0.41***	0.22***	0.24***	−0.09***

Note: gender is coded as 1=male and 2=female. Statistically significant estimates are in bold. **P<0.01, ***P<0.001.

**Table 2:** Results of the univariate GREML analysis for each personality trait and the unadjusted bivariate GREML analysis between all five personality traits

		*Bivariate correlations of SNP-*h^*2*^ *(s.e.)*			
*Personality trait*	*SNP-*h^*2*^*(s.e.)*	*1*	*2*	*3*	*4*
Extraversion	0.08 (0.08)	—			
Agreeableness	<0.001 (0.08)	<0.001 (<0.01)	—		
Conscientiousness	0.01 (0.08)	−0.31 (0.83)	<0.001 (<0.01)	—	
Neuroticism	**0.15 (0**.08)*	0.82 (0.39)*	<0.001 (<0.01)	−0.15 (0.73)	—
Openness	**0.21 (0**.08)**	0.46 (0.31)	<0.001 (<0.01)	−0.61 (1.01)	1.00 (0.50)***^a^

Abbreviations: GCTA, Genome-wide Complex Trait Analysis; GREML, genomic-relatedness-matrix residual maximum likelihood analysis; SNP, single-nucleotide polymorphism.

Note: the point estimates are reported to provide context for the s.e. and *P*-values. Statistically significant estimates are in bold.

a The raw correlation estimate was *rG*=1.25. Given that estimates of bivariate correlations in GCTA are not restricted to a range between −1 and 1, the correlation estimate of 1.25 can be interpreted as a correlation of 1 and was therefore capped at *rG*=1.00. *P<0.05, **P<0.01, ***P<0.001.
